# Nomogram for predicting cardiovascular mortality in patients with gastrointestinal stromal tumor: A population-based study

**DOI:** 10.1097/MD.0000000000039835

**Published:** 2024-09-27

**Authors:** Huimin Wang, Koulong Zheng, Chenhui Tai, Yimei Sun, Sujuan Feng, Yi Zhang, Ya-Dong Gao

**Affiliations:** a Department of Cardiology, Affiliated Hospital 2 of Nantong University, Nantong, China; b Nantong Clinical Medical College of Kangda College, Nanjing Medical University, Nantong, China; c Office of the Dean, Affiliated Hospital 2 of Nantong University, Nantong, China; d Department of Nephrology, Affiliated Hospital 2 of Nantong University, Nantong, China; e Department of Neurosurgery, Affiliated Hospital 2 of Nantong University, Nantong, China; f Department of Gastroenterology, Affiliated Hospital 2 of Nantong University, Nantong, China.

**Keywords:** cardiovascular death, competing-risk model, gastrointestinal stromal tumor, nomogram, SEER

## Abstract

This research aimed to develop and validate a clinical nomogram for predicting the probability of cardiovascular death (CVD) in patients with gastrointestinal stromal tumors (GIST). Information regarding patients diagnosed with GIST was extracted from the surveillance, epidemiology, and end results database. The multivariable competing risk model and multivariable Cox regression model were utilized to determine the independent predictive factors. A comparison was made between the results obtained from the 2 models. A nomogram was built to visualize the competing risk model. The nomogram’s performance was assessed utilizing concordance index, calibrate curve, decision curve analysis, and risk stratification. A total of 9028 cases were enrolled for final analysis, with CVD accounting for 12.8% of all deaths since GIST diagnosis. The multivariate analysis of competing risks revealed that age, chemotherapy and marital status were identified as independent risk factors for CVD in GIST individuals. The nomogram model exhibited good calibration and strong discriminative ability, indicating its effectiveness in predicting outcomes, with a concordance index of 0.788 (95% confidence interval: 0.753–0.823) in the training set, and 0.744 (95% confidence interval: 0.673–0.815) in the validation set. Decision curve analysis indicated that the prediction model had good clinical practicability. Additionally, risk stratification analysis efficiently divided GIST individuals into high- and low-risk populations for CVD. This was the first research to construct and validate a predictive nomogram using a competing risk model to estimate the individual probabilities of CVD in GIST patients. The nomogram can assist clinicians in making personalized treatment and monitoring plans.

## 1. Introduction

Gastrointestinal stromal tumors (GIST) are a common type of mesenchymal tumor found within the alimentary canal, comprising approximately 1% to 3% of all gastrointestinal malignancies.^[1]^ Epidemiological studies have shown that the incidence of GIST varied by patients’ geographic distribution, covering a range of 4.3 to 22 per million per year.^[2]^ Even though, most cases are cured by surgery if diagnosed early, more than 40% of GIST patients experience recurrence and metastasis, may leading to death.^[3]^ The overall survival time and time to distant metastasis following surgery in GIST patients have significantly improved due to timely diagnosis and effective treatments.^[4]^ Therefore, most causes of death are now attributed to noncancerous reasons rather than the cancer itself. Nevertheless, the risk of noncancerous death is a matter of concern that has not attracted enough attention from clinicians and society in general.

For the last few years, cardiovascular death (CVD) has long been considered to a common and serious side effect following cancer treatment.^[5,6]^ Studies have indicated that CVD mortality risk of cancer individuals remains increased compared to the general population.^[7]^ Indeed, the application of tyrosine kinase inhibitors (TKIs) as target therapies has significantly improved the survival rates of individuals with late GIST tumors.^[8]^ However, these drugs can also cause damage to non-cancer tissue,^[9]^ including the cardiovascular system,^[5,10]^ which sometimes lead to a variety of significant complications, especially with long-term courses. The side effects associated with GIST therapy, as well as tumor burden, may cause subsequent cardiovascular disease and events.^[11]^ As previously reported, CVD is the second most common cause of death in individuals with GIST diagnosed older than 80 years.^[12]^ Consequently, it is an emerging problem that requires intensifying awareness and research by experts from multiple disciplines, including cardiologists, immunologists, oncologists.

To achieve the most effective primary prevention or management strategies for CVD in the majority of GIST individuals, it is likely necessary to modify conventional risk factors. In clinical practice, identifying and stratifying the risk of CVD in patients with GIST is of utmost importance. This helps in making personalized decisions to optimize their management. As far as we know, there is only one published research on the cardiovascular outcomes in patients with GIST, which did not provide methods to assess the CVD risk in the individual patients with GIST.^[12]^ Although risk factors associated with CVD in individuals with GIST have been recognized, there is presently a lack of scoring systems available to predict CVD in this specific subpopulation. In this study, our aim was to identify risk factor for predicting CVD in GIST patients and construct an effective yet straightforward forecasting tool to estimate CVD risk for individual patients.

## 2. Methods

### 2.1. Study population and variables

As a population-based database, the surveillance, epidemiology, and end results (SEER) is updated annually. It serves as a malignant tumor registration system with 18 regional registries that cover approximately 28% of the American population.^[13]^ The SEER data were gathered and presented using the data items and codes specified by the North American Association of Central Cancer Registries.^[14]^ We identified patients in the up-to-date population-based database diagnosed with GIST between 2000 and 2019 by SEER*Stat software. The identification of GIST utilized the International Classification of Disease for Oncology code 8936 from the International Classification of Disease for Oncology, 3rd edition. The SEER database is a public database that removes personal identification, so this study does not require ethical board approval and patient informed consent.

**Figure 1.**
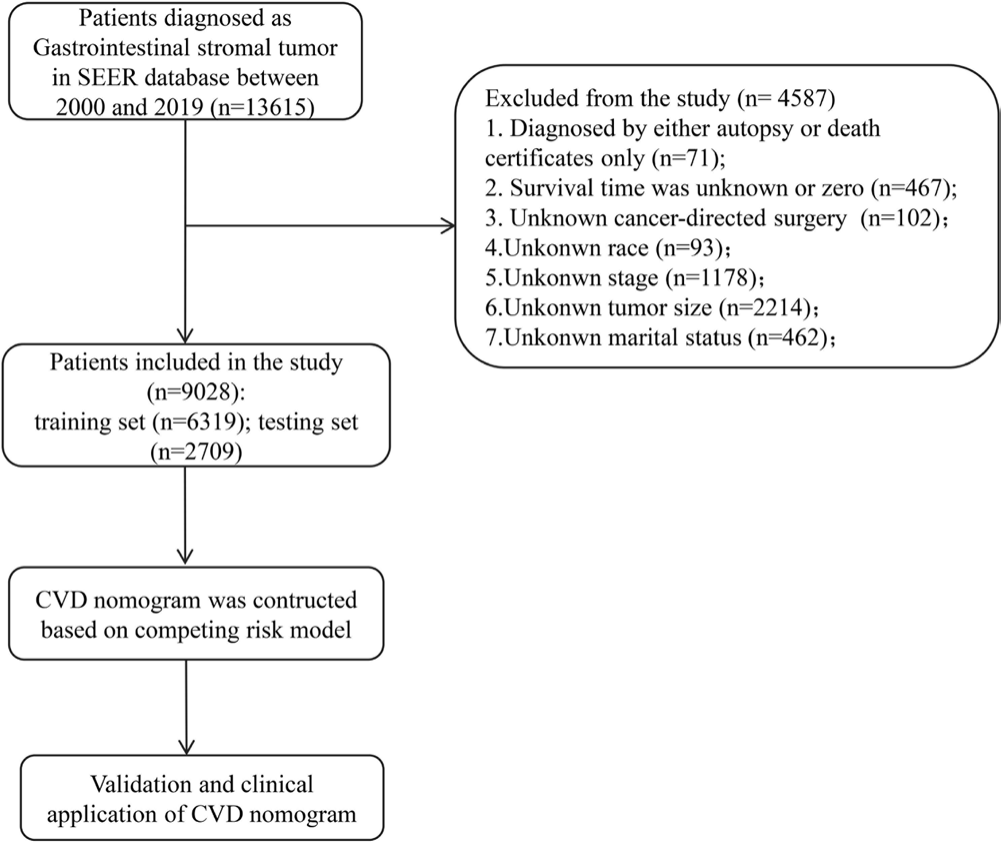
Study flowchart.

Figure 1 illustrates the process of selecting patients for the study, outlining the steps involved in their inclusion or exclusion. The exclusion criteria were: (1) Individuals who had cancer diagnosed through autopsy or death certificates were excluded from the selection process (N = 71); (2) patients whose survival time was unknown or zero were excluded (N = 467); (3) patients with unknown cancer-directed surgery were exclude (N = 102). Several demographic variables, including year of diagnosis, race, tumor site, sex, histological grade, SEER stage, surgery, chemotherapy, survival months, cause of death, age, tumor size, marriage were obtained from the population-based database. Individuals were classified as married, unmarried (including single, divorce, widowed, as well as separated) and unknown. Age was categorized into 4 groups.

### 2.2. Outcomes

The main outcome measure was CVD, which was assessed as the duration from GIST diagnosis until death related to cardiovascular disease. The causes of CVD recorded in the SEER data are as follows: diseases of heart, hypertension without heart disease, cerebrovascular diseases, atherosclerosis, aortic aneurysm and dissection as well as other diseases of arteries, arterioles, capillaries.^[15]^ The causes of death were divided into CVD and non-cardiovascular death (non-CVD). Non-CVD included deaths from cancers, as well as death from other and unknown causes that were not attributed to cardiovascular disease. Deaths attributed to non-CVD were considered as competing events for CVD. Individuals were regarded as censored if they lost during follow-up or were alive at the last follow-up.

### 2.3. Statistical analysis

All baseline characteristics were categorical variables or were converted into classified variables. These variables were described as frequencies (percentage) and were compared utilizing a Chi-square test. In our analysis, we treated death on account of other reasons as competing events. The cumulative incidence function (CIF) was utilized in univariate analyses to assess the probability of occurrence, and Gray test was employed to estimate the disparity in CIF between the groups.^[16]^ Multivariate analyses with the Fine-Gray subdistribution hazard competing risk regression were used to recognize the independent predictors of CVD among GIST patients.^[17]^ A Cox regression model was constructed and compared to the results from competing risk regression as a sensitivity analysis. In addition, a nomogram model was developed to predict the cumulative probabilities of CVD in GIST patients, based on significant factors identified in the multivariate competing risk model. We used Harrell concordance index (C-index) to assess the discrimination performance of the nomogram.^[18]^ A better C-index value indicated a more precise predictive ability. We also plotted calibration curves to measure the consistency of the model.^[19]^ In addition, decision curve analysis (DCA) was employed as a framework to improve the clinical utility value and benefits of the nomogram.^[20]^ The statistical software utilized for the analyses included IBM SPSS Statistics *V. 22.0* and R software *V. 3.6.1*. Several R packages, including *mstate*, *cmprsk*, *regplot*, *riskRegression*, *survival*, *pec*, as well as *foreign* were applied to develop and validate the nomogram. In two-sided tests, a statistically significant result was determined when the *P*-value was <.05. We followed standard *TRIPOD guidelines* for predicting models (see Fig. S1, Supplemental Digital Content, for details, http://links.lww.com/MD/N645).

## 3. Results

### 3.1. Clinical characteristics

Nine thousand twenty-eight patients who met the inclusion criteria between 2000 and 2019 in the SEER database were enrolled in our study. A summary of the demographic and clinical characteristics of the patients is presented in Table 1. The dataset was grouped into a training dataset (n = 6319) and an independent testing dataset (n = 2709) using a ratio of 7 to 3 in a random manner. There was no statistically significant difference observed between the training and testing datasets (*P* > .05). The majority of patients were aged above 60 years (63.3%), male (51.2%), white (68.1%), married (61.0%), had local SEER stage (68.4%), and tumor size ≥ 5 cm (59.3%). The stomach (61.7%) was identified as the most frequent location of the tumor, followed by the small intestine (27.1%). Moreover, low-grade tumors (grade I and II) took up a significantly larger percentage (25.7%) of cases compared to high-grade tumors (grade III and IV). The majority of the patients, 7654 (84.8%), opted for surgery as a treatment option, while in 3963 (43.9%) cases, chemotherapy was administered.

**Table 1:** Basic characteristics of the study populations and subpopulations.

Characteristics	Total (n = 9028)	Training dataset (n = 6319)	Validation dataset (n = 2709)	*P* value
**Age**				.850
＜40	455 (5.0)	311 (4.9)	144 (5.3)	
40–60	2858 (31.7)	2008 (31.8)	850 (31.4)	
60–80	4613 (51.1)	3224 (51.0)	1389 (51.3)	
≥80	1102 (12.2)	776 (12.3)	326 (12.0)	
**Sex**				.110
Female	4405 (48.8)	3118 (49.3)	1287 (47.5)	
Male	4623 (51.2)	3201 (50.7)	1422 (52.5)	
**Race**				.287
White	6150 (68.1)	4283 (67.8)	1867 (68.9)	
Non-White	2878 (31.9)	2036 (32.2)	842 (1867)	
**Marital status**				.242
Married	5506 (61.0)	3829 (60.6)	1677 (61.9)	
Unmarried	2878 (39.0)	2490 (39.4)	1032 (38.1)	
**Tumor site**				.662
stomach	5572 (61.7)	3925 (62.1)	1647 (60.8)	
small intestine	2451 (27.1)	1703 (27.0)	748 (27.6)	
Colorectum	387 (4.3)	267 (4.2)	120 (4.4)	
Other/unknown	618 (6.8)	424 (6.7)	194 (7.2)	
**Tumor size**				.795
＜5cm	3671 (40.7)	2575 (40.8)	1096 (40.5)	
≥5cm	5357 (59.3)	3744 (59.2)	1613 (59.5)	
**Grade**				.210
I/II	2318 (25.7)	1639 (25.9)	679 (25.1)	
III/IV	775 (8.6)	559 (8.8)	216 (8.0)	
unknown	5935 (65.7)	4121 (65.2)	1814 (67.0)	
**SEER stage**				.861
Localized	6172 (68.4)	4331 (68.5)	1841 (68.0)	
Regional	1243 (13.8)	866 (13.7)	377 (13.9)	
Distant	1613 (17.9)	1122 (17.8)	491 (18.1)	
**Surgery**				.884
No	1374 (15.2)	964 (15.3)	410 (15.1)	
Yes	7654 (84.8)	5355 (84.7)	2299 (84.9)	
**Chemotherapy**				.552
No	5065 (56.1)	3558 (56.3)	1507 (55.6)	
Yes	3963 (43.9)	2761 (43.7)	1202 (44.4)	

Others = American Islander India/Alaska Native/Asian/Pacific, SEER = surveillance, epidemiology, and end results.

### 3.2. CIF survival analysis

**Figure 2.**
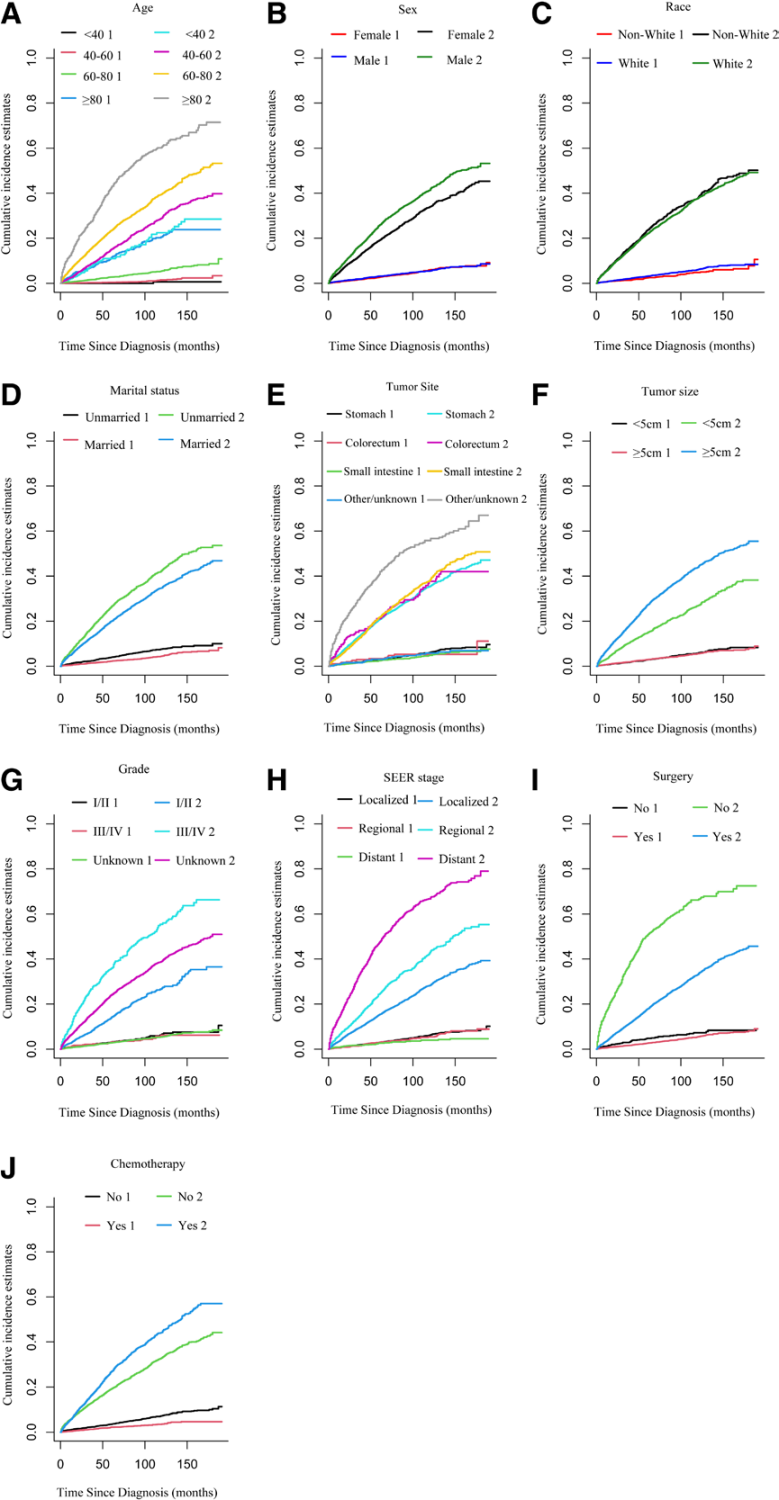
Cumulative incidence estimates for CVD in GIST patients stratified by (A) age; (B) sex; (C) race; (D) marital status; (E) tumor site; (F) tumor size; (G) grade; (H)stage; (I) surgery; (J) chemotherapy. CVD = cardiovascular death, GIST = gastrointestinal stromal tumor.

The median follow-up time for the entire population was 50 months (inter-quartile range: 21.00–93.00). A total of 2678 deaths (29.7%) were observed, with 343 deaths (12.8%) from CVD and 2335 deaths (87.2%) from non-CVD. The univariate analysis included the CIF and Gray test. To account for competing risks (other causes of death), we subjected the whole cohort to cumulative incidence analysis (Table 2). In general, the CIF for 1, 3, and 5 years CVD were 0.73%, 1.79%, and 2.90%, respectively, corresponding to non-CVD of 5.64%, 14.3%, and 22.4%. Furthermore, we perform subgroup analysis stratified by all variables (Table 2). A higher incidence of CVD was observed in individuals aged ≥ 80 years (Fig. 2A) who were of White ethnicity (Fig. 2C), unmarried (Fig. 2D); who did not undergo surgery (Fig. 2I) or receive chemotherapy (Fig. 2J). Furthermore, we did not find any significant differences in CVD based on sex (Fig. 2B), tumor site (Fig. 2E), tumor size (Fig. 2F), localized stage (Fig. 2H), and grade (Fig. 2G) subgroup analysis.

**Table 2:** Cumulative incidence and Gray test of cause-specific death in the whole set.

Characteristics	CVD(%)			*P*	Non-CVD(%)			*P*
	1-year	3-year	5-year		1-year	3-year	5-year	
Total	0.73	1.79	2.90		5.64	14.3	22.41	
**Age**				<.001				<.001
＜40	<0.001	<0.001	<0.001		2.06	7.67	10.98	
40–60	0.11	0.32	0.43		2.48	8.67	15.04	
60–80	0.63	1.59	2.73		6.31	15.36	23.39	
≥80	3.10	7.16	11.32		12.50	27.39	42.43	
**Sex**				.686				<.001
Female	0.56	1.70	2.67		4.67	12.03	19.00	
Male	0.90	1.87	3.11		6.57	16.49	25.60	
**Race**				.045				.195
White	0.70	1.96	3.10		5.57	13.99	21.92	
Non-White	0.80	1.40	2.43		5.79	15.06	23.49	
**Marital status**				<.001				<.001
Married	0.51	1.42	2.22		4.99	12.78	19.91	
Unmarried	1.09	2.35	3.95		6.65	16.73	26.28	
**Tumor site**				.36				<.001
stomach	0.72	1.78	3.06		5.02	13.32	20.66	
small intestine	0.67	1.68	2.42		4.33	12.42	21.67	
Colorectum	1.05	2.92	3.32		7.84	15.88	21.09	
Other/unknown	0.85	1.56	3.08		15.05	29.53	40.77	
**Tumor size**				.715				<.001
＜5cm	0.68	1.75	2.86		3.33	9.94	14.83	
≥5cm	0.77	1.81	2.92		7.20	17.20	27.16	
**Grade**				.714				<.001
I/II	0.78	2.00	3.07		2.61	7.79	13.97	
III/IV	0.91	2.10	3.12		10.10	26.67	35.80	
unknown	0.69	1.65	2.79		6.27	15.34	23.96	
**SEER Stage**				.050				<.001
Localized	0.74	1.87	2.96		3.41	9.44	15.29	
Regional	0.57	1.47	2.92		5.91	14.54	24.37	
Distant	0.83	1.72	2.59		13.97	32.74	47.27	
**Surgery**				.005				<.001
No	1.29	2.94	4.78		17.02	35.66	50.32	
Yes	0.64	1.59	2.58		3.63	10.64	17.71	
**Chemotherapy**				<.001				<.001
No	0.98	2.24	3.48		5.67	12.86	19.11	
Yes	0.42	1.20	2.15		5.60	16.23	26.75	

CVD = cardiovascular death, others = American Islander India/Alaska Native/Asian/Pacific, SEER = surveillance, epidemiology, and end results.

### 3.3. Multivariate analysis on prognosis of GIST

As shown in Table 1, the cohort included 9028 patients with GIST, with 6319 patients in the training set for nomogram construction and 2709 patients in the validation set for internal verification. All the characteristics/variables in the 2 groups were not statistically different. To determine the independent predictors in the training set, we incorporated all statistically significant factors identified in the univariate analysis into the Fine-Gray competing risks model. As shown in Table 3, after adjusting for all possible confounders, there were finally 3 parameters significantly correlated with CVD, age (60–80: sub-distribution hazard ratio [sdHR] 14.40, 95% confidence interval [CI]: 1.99–104.12, *P* = .008; ≥80: 74.22, 10.28–535.74, *P* < .001), marital status (married: 0.72, 0.55–0.94, *P* = .015), and chemotherapy (yes: 0.67, 0.50–0.90, *P* = .008). Subsequently, multivariate Cox regression was performed for comparison. The Cox regression analysis revealed that age (60–80: hazard ratio [HR] 14.60, 95% CI: 2.03–104.78, *P* = .008; ≥80: 77.75, 10.81–559.12, *P* < .001), surgery (yes: 0.68, 0.47–0.98, *P* = .040), marital status (married: 0.72, 0.55–0.94, *P* = .014), and chemotherapy (yes: 0.67, 0.50–0.90, *P* = .008) were independent predictors.

**Table 3:** Results of multivariate analysis by the Fine-Gray and Cox models in training cohort.

Variables	Fine-Gray		Cox	
	sdHR (95%CI)	*P*	HR (95%CI)	*P*
**Age**				
＜40	Reference		Reference	
40–60	3.58 (0.48–26.90)	.215	3.59 (0.48–26.74)	.212
60–80	14.40 (1.99–104.12)	.008	14.60 (2.04–104.78)	.008
≥80	74.22 (10.28–535.74)	<.001	77.75 (10.81–559.13)	<.001
**Race**				
Non-White	Reference		Reference	
White	1.18 (0.87–1.60)	.277	1.18 (0.87–1.59)	.285
**Marital status**				
Unmarried	Reference		Reference	
Married	0.72 (0.55–0.94)	.015	0.72 (0.55–0.94)	.014
**Surgery**				
No	Reference		Reference	
Yes	0.73 (0.50–1.07)	.107	0.69 (0.48–0.98)	.041
**Chemotherapy**				
No	Reference		Reference	
Yes	0.67 (0.50–0.90)	.008	0.67 (0.50–0.90)	.008

CI = confidence interval, CVD = cardiovascular death, GIST = gastrointestinal stromal tumor, HR = hazard ratio, Others = American Islander India/Alaska Native/Asian/Pacific, sdHR = subdistribution hazard ratio, SEER = surveillance, epidemiology, and end results.

### 3.4. Establishment and verification of competing-risk model

**Figure 3.**
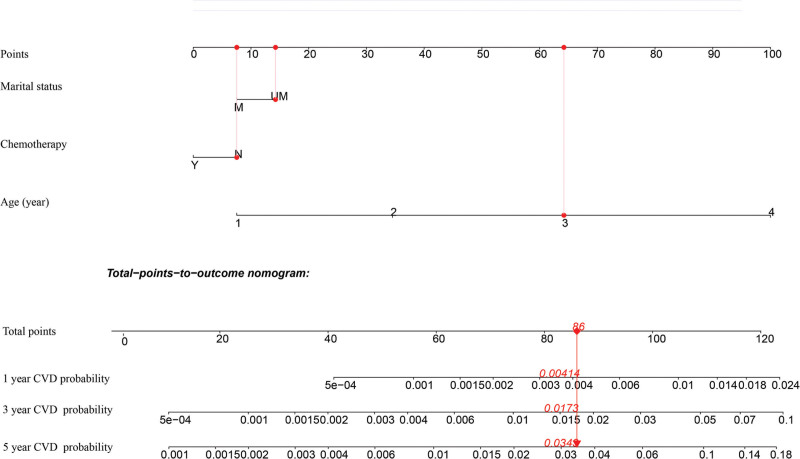
Postoperative nomogram for predicting 1-, 3- and 5-year probabilities of CVD in GIST patients. CVD = cardiovascular death, GIST = gastrointestinal stromal tumor.

**Figure 4.**
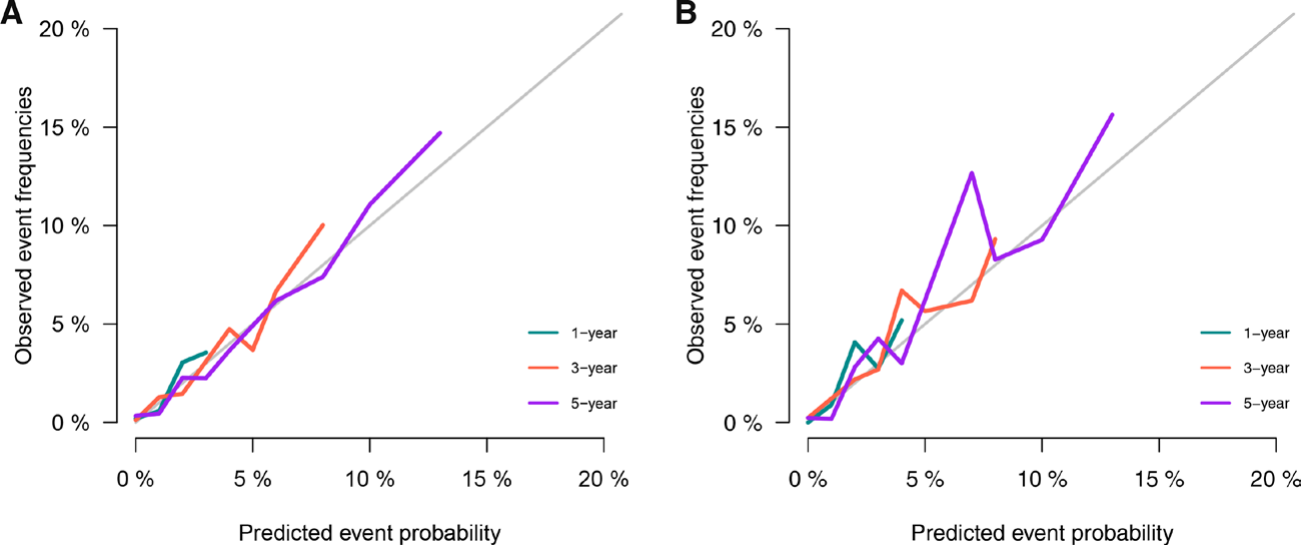
Calibration curves for predicting CVD in the training cohort (A) and the validation cohort (B), respectively. CVD = cardiovascular death.

**Figure 5.**
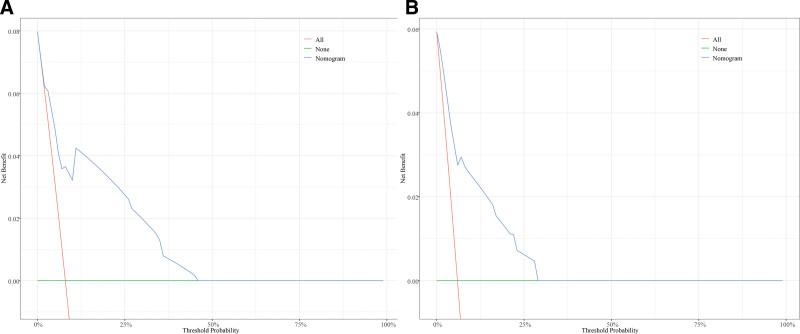
Decision curve analysis of CVD nomogram at 1-, 3-, and 5-year in the training cohort (A) and validation cohort (B). CVD = cardiovascular death.

Based on Fine-Gray competing risk model, we built a nomogram to predict the risk of CVD (Fig. 3). Three significant variables, including age, marital status, and chemotherapy, were including in the nomogram. For the nomogram predicting CVD, age was found to be the strongest predictor, followed by marital status and history of chemotherapy. The corresponding probabilities of CVD could easily be evaluated by calculating the total score of each variable. Our nomogram demonstrated good discrimination and predictability. In the training cohort, our model achieved a C-index estimate of 0.788 (95% CI: 0.753–0.823). Meanwhile, when evaluated on the validation cohort, the C-index was slightly lower at 0.744 (95% CI: 0.673–0.815). The calibration curves for the training and validation datasets, indicating the predicted probabilities of CVD at 1, 3, and 5 years, were presented in Figure 4. All data points were observed to be in close proximity to the standard 45-degree diagonal line, suggesting a strong correspondence between the predicted CVD rates and the actual CVD rates. Furthermore, based on the threshold probability, our study adopted DCA to prove the best predictive model, making the results more suitable for clinical application. DCA demonstrated that the application of nomogram model provided a higher net benefit in predicting CVD rates across a wide range of threshold probability (Fig. 5A and B), which suggested exhibited significant clinical utility in predicting CVD.

**Figure 6.**
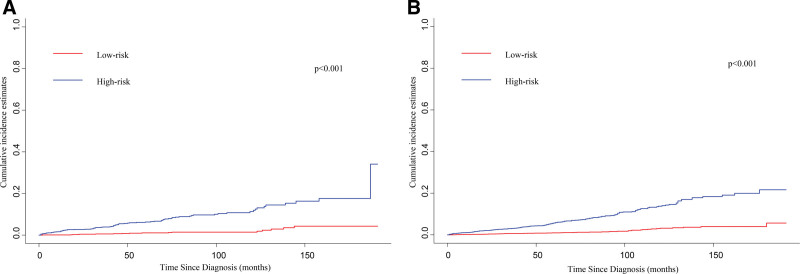
CIF curves with the *P*-value of Gray test of the stratified risk groups in the training cohort (A) and validation cohort (B). CIF = cumulative incidence function.

Subsequently, in accordance with the median (68.22) of the nomogram scores, GIST individuals were classified into low-risk and high-risk population in both the training and validation sets. Patients in high-risk groups had higher likelihood of CVD in each cohort (Fig. 6A and B). Collectively, the findings suggest that the competing risk model provides a valuable tool for clinical risk assessment and decision-making for individuals diagnosed with GIST.

## 4. Discussion

Despite the adoption of early tumor screening and considerable advances in tumor therapies that have significantly improved the survival rates for patients with GIST in the past several years, non-cancer deaths still contribute significantly to GIST patient outcomes, primarily due to cardiovascular disease. Utilizing the SEER database, our present study had yielded significant findings regarding the risk factors for cardiovascular issue in patients diagnosed with GIST between 2000 and 2019. During the follow-up period, a total of 2678 patients (29.7%) experienced mortality, with 343 individuals (3.8%) specifically succumbing to CVD. The CIF for non- CVD over a period of 1, 3, and 5 years were 5.64%, 14.3%, and 22.4%, respectively. In addition, for CVD, the corresponding CIF during the same time frame were 0.73%, 1.79%, and 2.90%, suggest that CVD have a significant impact on the mortality of patients diagnosed with GIST.

Independent predictors of CVD, including age, marital status, surgery, and history of chemotherapy, were identified for GIST patients using the Cox and Kaplan–Meier model. However, after adjustment by competing-risk regression model, factors that affected the probability of CVD were age, marital status, and chemotherapy, unlike the results obtained from the traditional Cox and Kaplan–Meier analysis. The conventional analytical approach, when examining a particular cause of death, treated competing events as censored observations and ignored the impact of competing events tends to result in an overestimation of the cumulative incidence associated with each variable. The Cox regression model overestimated the risk of surgery. Initially, the Cox model demonstrated a significant association between surgery and a lower incidence of CVD in GIST patients (HR 0.69, 95% CI: 0.48–0.98, *P* = .041). However, the Fine-Gray model did not find a statistically significant effect of surgery on CVD, with a sdHR of only 0.73. According to Cox regression analysis, individuals aged 60–80 (HR 14.60, 95% CI 2.04–104.78, *P* = .008) and age ≥ 80 years (HR 77.75, 95% CI: 10.81–559.13, *P* < .001) were found to have higher risks of CVD in comparison to those aged < 60 years with GIST, and the Fine-Gray regression model, in contrast to the Cox regression model, demonstrated that the risk of age was overestimated (60–80: sdHR 14.40, 95% CI: 1.99–104.12, *P* = .008; ≥80: 74.22, 10.28–535.74, *P* < .001). A significant limitation in traditional survival analyses, such as Cox regression and Kaplan–Meier methods, is the treatment of competing risks—specifically, non-cancer-specific deaths—as censored data. This approach overlooks the impact of non-cancer-specific mortality on survival outcomes, which can lead to misunderstandings or biases in the analysis results.^[21]^ Traditional survival analyses, such as Cox regression and Kaplan–Meier methods, often face significant limitations due to their treatment of competing risks, particularly non-cause-specific deaths (NCSD), as censored data. This practice neglects the influence of non-cancer-specific mortality on survival outcomes, potentially leading to misinterpretations or biases in the results.^[22]^ Therefore, for more accurate results, it is advisable to utilize competing risk models when there are one or more competing risks, particularly within middle-aged and older populations.^[23]^

For effective patient consultation and to optimize individualized clinical decision-making, it is crucial to conduct a thorough assessment of the prospective risk for cardiac toxicity in each individual. we have achieved success in developing and validating a model for assessing the risk of CVD in newly diagnosed GIST patients. This model incorporates 3 baseline variables and takes into account competing risks. To the best of our knowledge, this groundbreaking study is the first to employ a Fine-Gray sub-distribution hazard model for constructing a competing risk analysis. This unique approach enables us to accurately estimate the probability of CVD-specific mortality in patients diagnosed with GIST. All the variables, which were routinely collected in clinical practice, and were easily available, have been included in our predictive nomogram. Besides, the reproducibility of our simple and standardized nomogram was excellent. Its ability to accurately identify patients at high risk of CVD and guide clinical decision-making proved to be effective. Clinicians can rely on this tool to optimize their decision-making process and enhance patient management. Furthermore, it is crucial to remain vigilant and exercise caution while treating patients with GIST in order to address this potential complication effectively. The risk of CVD can be predicted on an individual basis using this user-friendly scoring model, making it accessible to healthcare professionals, the general public, and patients with minimal training. By utilizing the nomogram, we can determine the cumulative risk score for an individual aged 60–80 (65 points), who is not married (15 points), and has not undergone chemotherapy (6 points). In this case, the total point score amounts to 86. This equates to 1, 3, and 5 years CVD rates of 0.414%, 1.73%, 3.43%, respectively. We validated the accuracy and discrimination of our nomograms through C-index assessment and calibration curves, which demonstrated satisfactory results. To evaluate the practicality of the developed nomograms, we conducted DCA and calculated net benefits across various threshold probabilities.^[24]^ After verification, DCA revealed that our nomograms have good clinical value and utility in forecasting the likelihood of CVD. Taken together, the model had high reliability and strong practicability, making it suitable for clinical promotion.

Recently, the role of socioeconomic status in human health, including income, education attainment, employment and insurance status, as well as marriage status, has attracted increasing attention in the global medical community.^[25,26]^ The impact of marriage on the survival of patients with various malignancies has been shown to be positive. However, its effect may vary by not only quantity and quality of research but also cancer type.^[27–29]^ In the present study, we discovered that the marital status of patients in this population was a separate prognostic factor for CVD. Our results have shown that the incidence of CVD at 1, 3, and 5 years was consistently lower among married patients compared to those who were widowed, single, separated, or divorced, irrespective of age and whether they treated with chemotherapy or not. Previous studies indicated that married patients, who are more likely to have better mental status and receive encouragement and help from their spouse and society, have better compliance with prescribed therapy.^[29]^ Married patients, who often possess greater social and economic resources, are more likely to lead healthier lifestyles and acquire early screening and accurate healthcare services.^[27,30]^ In addition, from a biological standpoint, marital status has a positive effect on cardiovascular and immune function, cortisol level and endocrine function, which may benefit cancer treatment and management.^[29,31]^ We therefore strongly suggest the integration of socioeconomic factors, particularly marital status, when assessing individual risk of CVD in GIST survivors.

In addition to marital status, age is another independent risk factor for CVD. It is to be expected that cardiovascular mortality risk is higher among elderly patients in comparison to younger patients diagnosed with GIST. As age advances, patients commonly have decreased immunity, increased comorbidities,^[32]^ accelerated atherosclerosis, thrombophilic status associated with malignancy and postoperative status, and fewer physiological reserves combined with more impaired cardiovascular function. These factors could potentially impact the American Society of Anesthesiologists physical status classification, which may later induce a higher perioperative CVD risk, an extended recovery period postoperatively, and greater cardiotoxicity after treatment with cytotoxic chemotherapy.

The sensitivity of most GISTs with tyrosine kinase receptor or platelet-derived growth factor receptor-alpha mutations to TKIs (including imatinib, sunitinib, regorafenib, and so on) has been approved.^[33–35]^ Worryingly, the administration of imatinib, a type of TKI, has been linked to an elevated risk of CVD due to its cardiotoxic effects.^[36,37]^ Moreover, individuals undergoing chemotherapy face a greater susceptibility to CVD than the general population.^[12]^ However, our analysis showed that GIST patients who were treated with chemotherapy have significantly lower risks of CVD compared to those without chemotherapy. Our research had produced a seemingly contradictory discovery. It was worth mentioning that this outcome aligned with previous studies conducted on GIST^[12]^ and other types of tumors such as colorectal cancer,^[38]^ primary central nervous system lymphoma,^[39]^ and bladder cancer.^[40]^ There were several factors that might explain this connection. Firstly, the SEER database had numerous cases with missing treatment data, including information about the patient’s chemotherapy regime, duration, and whether they had co-existing CVD at the time of diagnosis. Individuals with preexisting CVD are not suitable candidates for chemotherapy, and those with better cardiovascular health are more likely to receive it.^[41]^ Consequently, the absence of chemotherapy resulted in a higher risk of cardiovascular death. Secondly, chemotherapy is commonly used for metastatic GIST and cases where resection of GIST is not possible. Thus, individuals with a limited life expectancy and a higher likelihood of dying from GIST itself are more likely to receive chemotherapy. However, the underlying mechanism behind this phenomenon remains unclear, necessitating further investigation in the future.

Our analysis had several strengths. First, the basis of our study relied on the SEER database, which provided a large enough sample data and was collected from a multicenter population to enhance the representativeness of the findings. Second, as stated in a cardio-oncology guideline published in 2022,^[42]^ it is recommended that all cancer patients who were scheduled to receive potentially cardiotoxic anticancer therapy undergo a baseline cardiovascular risk assessment. This will help the oncology teams in making treatment decisions for cancer and allow them to create individualized strategies for monitoring and follow-up of cardiovascular health. However, there are currently no risk stratification tools to help evaluate those at risk of CVD in GIST patients. For these patients, we developed the first nomogram for individualized prognosis prediction. It is vital to focus on prevention, monitoring, and intervention of cardiovascular complications in individuals who are at a high risk of cardiovascular death. Third, the nomograms were of practical utility because all clinical variables included in the nomogram model were readily available and commonly collected, and individual CVD calculations could be completed in <3 minutes. Finally, our nomogram showed excellent performance in discriminatory ability and clinical availability.

we acknowledge several limitations of this study. First, it was a retrospective study. Systematic, as well as prospective data, were lacking, which might possibly lead to selection bias. Second, the SEER registry did not provide important risk factors influencing the risk of CVD, such as comorbidity diseases, personal life history of smoking and alcohol use, and diet. Third, comprehensive information regarding chemotherapy was not provided by the SEER registry. Additionally, although the good performance of our nomogram was internally validated, we should carry out external validation in large-scale prospective clinical trials in the future. These trials will provide ample opportunities to further evaluate the robustness and generalizability of the nomogram.

## 5. Conclusions

We made the first attempt to develop and validate a predictive nomogram. This groundbreaking approach involved utilizing a competing risk model to estimate individual probabilities of CVD in GIST patients. The nomogram exhibited relatively satisfactory predictive performance and was well demonstrated in the internal validation. This well-established nomogram could provide critical guidance for clinicians and policymakers to estimate CVD probabilities of GIST, identify high-risk patients, make more precise and personalized therapies, and develop individual monitoring plans.

## Acknowledgments

The findings of this study are supported by data available from the Surveillance, Epidemiology, and End Results (SEER) program. In addition, the invaluable support of ChatGPT was instrumental in enhancing sentence structure revisions.

## Author contributions

**Data curation:** Koulong Zheng, Yimei Sun.

**Funding acquisition:** Yimei Sun, Sujuan Feng, Yi Zhang, Ya-Dong Gao.

**Methodology:** Chenhui Tai.

**Resources:** Chenhui Tai.

**Visualization:** Sujuan Feng, Yi Zhang.

**Writing – original draft:** Huimin Wang.

**Writing – review & editing:** Huimin Wang, Ya-Dong Gao.

## Supplementary Material


