# Supplementary material for: Nomogram for predicting cardiovascular mortality in patients with gastrointestinal stromal tumor: A population-based study
Source: Medicine (Baltimore). 2024 Sep 27;103(39):e39835. doi: 10.1097/MD.0000000000039835 (PMC11441931; doi:10.1097/MD.0000000000039835)
Supplement: Supplementary file 1 [file medi-103-e39835-s001.pdf]

Supplementary Figure 1. TRIPOD Checklist for prediction model development and validation with added text excerpts.

Section/Topic	Item		Checklist Item	Page	Text Excerpt
**Title and abstract**					
Title	1	D;V	Identify the study as developing and/or validating a multivariable prediction model, the target population, and the outcome to be predicted.	1	"Nomogram for predicting cardiovascular mortality in patients with gastrointestinal stromal tumor: a population-based study"
Abstract	2	D;V	Provide a summary of objectives, study design, setting, participants, sample size, predictors, outcome, statistical analysis, results, and conclusions.	2	OBJECTIVE/ STUDY DESIGN/ SETTING/ PARTICIPANTS/ OUTCOME: "This research aimed to develop and validate a clinical nomogram for predicting the probability of cardiovascular death (CVD) in patients with Gastrointestinal stromal tumors (GIST)."SAMPLE SIZE: "N total=9028" PREDICTORS: "The prediction models included age, chemotherapy and marital status". STATISTICAL ANALYSIS: "Information regarding patients diagnosed […]as risk stratification ." RESULTS: "A total of 9028 cases […] populations for CVD." CONCLUSIONS: "This was the first research […] and monitoring plans."
**Introduction**					
Background and objectives	3a	D;V	Explain the medical context (including whether diagnostic or prognostic) and rationale for developing or validating the multivariable prediction model, including references to existing models.	2/3	"Gastrointestinal stromal tumors (GIST) […] immunologists, oncologists."
	3b	D;V	Specify the objectives, including whether the study describes the development or validation of the model or both.	3	"To achieve the most effective primary […] risk for individual patients."
**Methods**					
Source of data	4a	D;V	Describe the study design or source of data (e.g., randomized trial, cohort, or registry data), separately for the development and validation data sets, if applicable.	3	"As a population-based database […] American population."
	4b	D;V	Specify the key study dates, including start of accrual; end of accrual; and, if applicable, end of follow-up.	3	"between 2000 and 2019 by SEER*Stat software"
Participants	5a	D;V	Specify key elements of the study setting (e.g., primary care, secondary care, general population) including number and location of centres.	3	We included all "patients diagnosed with GIST by SEER*Stat software."
	5b	D;V	Describe eligibility criteria for participants.	3	"The identification of GIST utilized the International Classification of Disease for Oncology (ICD-O-3) code 8936 from the ICD-O-3, 3rd edition. The exclusion criteria were: (1) Had cancer diagnosed through autopsy or death certificates ; (2) Whose survival time was unknown or zero; (3) With unknown cancer-directed surgery."
	5c	D;V	Give details of treatments received, if relevant.	NA	NA
Outcome	6a	D;V	Clearly define the outcome that is predicted by the prediction model, including how and when assessed.	4	"The main outcome measure was CVD, which was assessed as theduration from GIST diagnosis until death related to cardiovascular disease."
	6b	D;V	Report any actions to blind assessment of the outcome to be predicted.	NA	NA
Predictors	7a	D;V	Clearly define all predictors used in developing or validating the	5	"As shown in Table 1, […] were independent predictors"

			multivariable prediction model, including how and when they were measured.		
	7b	D;V	Report any actions to blind assessment of predictors for the outcome and other predictors.	NA	NA
Sample size	8	D;V	Explain how the study size was arrived at.	4/5	Method section: "We identified patients in […] in a random manner. "Result section: "9028 patients who […] presented in Table 1."
Missing data	9	D;V	Describe how missing data were handled (e.g., complete-case analysis, single imputation, multiple imputation) with details of any imputation method.	4	"Individuals were regarded as censored if they lost during follow-up or were alive at the last follow-up."
Statistical analysis methods	10a	D	Describe how predictors were handled in the analyses.	4	"The cumulative incidence function [...] competing risk model."
	10b	D	Specify type of model, all model-building procedures (including any predictor selection), and method for internal validation.	NA	NA
	10c	V	For validation, describe how the predictions were calculated.	4	"The cumulative incidence function […] multivariate competing risk model"
	10d	D;V	Specify all measures used to assess model performance and, if relevant, to compare multiple models.	4	We used Harrell′s concordance index […] benefits of the nomogram(20,21)."
	10e	V	Describe any model updating (e.g., recalibration) arising from the validation, if done.	NA	NA
Risk groups	11	D;V	Provide details on how risk groups were created, if done.	6	"Subsequently, in accordance with […] in each cohort (Figure 6A and B)."
Development vs. validation	12	V	For validation, identify any differences from the development data in setting, eligibility criteria, outcome, and predictors.	14	Difference in setting, outcome and predictors are described in Table 1.
**Results**					
Participants	13a	D;V	Describe the flow of participants through the study, including the number of participants with and without the outcome and, if applicable, a summary of the follow-up time. A diagram may be helpful.	17/Fig 1/5	Overview of selection of IPD: Figure 1<br>Participants: "9028 patients who […] was administered."
	13b	D;V	Describe the characteristics of the participants (basic demographics, clinical features, available predictors), including the number of participants with missing data for predictors and outcome.	5/Table 1	"9028 patients who met […] chemotherapy was administered."
	13c	V	For validation, show a comparison with the development data of the distribution of important variables (demographics, predictors and outcome).	Table 1	As shown in Table 1, the cohort included 9028 patients with GIST, with 6319 patients in the training set for nomogram construction and 2709 patients in the validation set for internal verification.
Model development	14a	D	Specify the number of participants and outcome events in each analysis.	NA	NA
	14b	D	If done, report the unadjusted association between each candidate predictor and outcome.	NA	NA
Model specification	15a	D	Present the full prediction model to allow predictions for individuals (i.e., all regression coefficients, and model intercept or baseline survival at a given time point).	6	Based on Fine-Gray competing risk model
	15b	D	Explain how to the use the prediction model.	7	By utilizing the nomogram [...] 0.414%, 1.73%, 3.43%, respectively.
Model performance	16	D;V	Report performance measures (with CIs) for the prediction model.	13/14	Reported
Model-updating	17	V	If done, report the results from any model updating (i.e., model specification, model performance).	NA	NA
**Discussion**					
Limitations	18	D;V	Discuss any limitations of the study (such as nonrepresentative sample, few events per predictor, missing data).	20	"A potential limitation […] with the primary aim of our study."

Interpretation	19a	V	For validation, discuss the results with reference to performance in the development data, and any other validation data.	6	“In the training cohort […] in each cohort (Figure 6A and B).”
	19b	D;V	Give an overall interpretation of the results, considering objectives, limitations, results from similar studies, and other relevant evidence.	6	“Despite the adoption of early […]patients diagnosed with GIST.”
Implications	20	D;V	Discuss the potential clinical use of the model and implications for future research.	7/8	“For effective patient consultation […] making it suitable for clinical promotion.”
**Other information**					
Supplementary information	21	D;V	Provide information about the availability of supplementary resources, such as study protocol, Web calculator, and data sets.	Suppleme nts S1	TRIPOD Checklist for prediction model development and validation with added text excerpts.
Funding	22	D;V	Give the source of funding and the role of the funders for the present study.	10	“This work was supported […] Province (MS2021060).”

*Items relevant only to the development of a prediction model are denoted by D, items relating solely to a validation of a prediction model are denoted by V, and items relating to both are denoted D;V. We recommend using the TRIPOD Checklist in conjunction with the TRIPOD Explanation and Elaboration document.
